# Impact of the SARS-CoV-2 pandemic and associated lockdown measures on attendances at emergency departments in English hospitals: A retrospective database study

**DOI:** 10.1016/j.lanepe.2021.100034

**Published:** 2021-01-13

**Authors:** Steven Wyatt, Mohammed A Mohammed, Elizabeth Fisher, Ruth McConkey, Peter Spilsbury

**Affiliations:** a The Strategy Unit, NHS Midlands and Lancashire Commissioning Support Unit, . Kingston House, 438-450 High Street, West Bromwich, West Midlands B70 9LD, UK; b The Strategy Unit, NHS Midlands and Lancashire Commissioning Support Unit, West Bromwich, West Midlands UK; c Faculty of Health Studies, University of Bradford, Bradford, UK; d The Nuffield Trust, London, UK; e The Health Foundation, London, UK; f The Strategy Unit, NHS Midlands and Lancashire Commissioning Support Unit, West Bromwich, West Midlands UK

## Abstract

**Background:**

The SARS-CoV-2 outbreak and associated lockdown measures have challenged healthcare. We examine how attendances to ED in England were impacted.

**Methods:**

Interrupted time series regression (January 2019 to June 2020) of data from EDs in 41 English NHS Trusts was used to estimate the initial decrease in attendances and the rate of increase following an interruption from 11 March – 7 April 2020, which included the 23 March lockdown in England.

**Findings:**

The SARS-CoV-2 interruption led to an initial 51.1% reduction (95% CI 46.3–55.9%) in ED attendances followed by a linear increase in attendances of 3.0% per week (95% CI 2.5–3.5%).  Significantly larger initial reductions were seen in those aged 0–19 years (69.1%), Indian (64.9%), Pakistani (71.8%), Bangladeshi (75.3%), African (63.5%) and Chinese people (74.5%), self-conveying attendees (60.3%) and those presenting with contusions or abrasions (66.9%), muscle and tendon injuries (65.6%), and those with a diagnosis that was not classifiable (72.7%).  Significantly smaller initial reductions were seen in those aged 65–74 years (42.6%), 75+ years (40.1%), those conveyed by ambulance (31.9%), and those presenting with the following conditions: central nervous system (44.9%), haematological (44.0%), cardiac (43.7%), gastrointestinal (43.4%), gynaecological (43.2%), psychiatric (40.4%), poisoning (39.7%), cerebro-vascular (39.0%), endocrinological (36.1%), other vascular (34.6%), and maxillo-facial (19.7%). No significant differences in the initial reduction of activity were seen in subgroups defined by sex, deprivation, urbanicity or acuity.

**Interpretation:**

The SARS-CoV-2 outbreak and lockdown substantially reduced ED activity. The reduction varied by age groups, ethnicity, arrival mode and diagnostic group but not by sex, deprivation, urbanicity or acuity.

**Funding:**

No funding to declare.

**Research in context**

**Evidence before this study**

Earlier studies have reported that the SARS-CoV-2 outbreak and associated ‘lockdown’ measures were associated with reductions in paediatric ED attendances, admissions for acute cardiac syndromes, face-to-face GP consultations and childhood immunisations.

**Added value of this study**

This study provides a more detailed assessment of the impact on attendance rates across a large sample of EDs, reporting results by age, gender, ethnicity, deprivation, urbanicity, arrival mode, acuity, and diagnosis. The analysis quantifies the immediate reductions in activity, the subsequent rate of increase and includes estimates of uncertainty.

**Implications of all the available evidence**

There were substantial reductions in ED attendances for all patient subgroups assessed and for most presenting diagnoses. Further work is required to understand the extent to which these activity reductions have or will lead to patient harm.

Alt-text: Unlabelled box

## 1 Introduction

The severe acute respiratory syndrome coronavirus 2 (SARS-CoV-2) outbreak, which was first reported in Wuhan China in December 2019 [1], has posed a major challenge to governments and healthcare providers all over the world. Early evidence indicated the possibility of human-to-human transmission and a higher risk of mortality predominantly in older people from the subsequent disease known as COVID-19 [2].

As part of the response to the pandemic, governments implemented a range of non-pharmaceutical interventions which included physical distancing measures, such as closures of schools, retail businesses, and restaurants, as well as restrictions on individual movements and social interactions [3,4]. Collectively these non-pharmaceutical interventions have come to be known as ‘lockdown’ measures.

The first two cases of COVID-19 in the UK were confirmed on 31 Jan 2020 [5]. The World Health Organisation (WHO) declared a pandemic on the 11 March 2020 and on the 23 of March 2020 the UK Government imposed measures which placed significant restrictions on its people [6] to reduce the transmission of SARS-CoV-2 [7].Health protection regulations were introduced on 26 March 2020 [8]. Members of the public were instructed to leave their homes for very limited purposes, including (1) shopping for basic necessities, for example food and medicine, as infrequently as possible, (2) one form of exercise a day, for example a run, walk, or cycle—alone or with members of their household, (3) any medical need, or to provide care or to help a vulnerable person, and (4) travelling for work purposes, but only where this cannot be done from home. Failure to comply with these rules could result in a fine or arrest. When people did leave the house, they must stay at least two meters away from people who do not live in their household. In March 2020, patients with possible COVID-19 symptoms were told to self-isolate, to contact NHS111, a free-to-call medical helpline, if they were concerned about their condition but not to go to their GP, pharmacy, or hospital [9]. A campaign to urge people to seek urgent care for other conditions when they need it was launched on 25th of April 2020 [10].

We systematically examine the impact of SARS-CoV-2 and the associated lockdown measures on levels of ED activity in England by adopting an interrupted time series regression approach with a 4-week interruption period containing the introduction of the lockdown measures in the UK [7] on 23rd March 2020. We also examine the extent to which the impact of the interruption varied by patient characteristics, such as age, sex, deprivation, ethnicity, mode of presentation and clinical presentation.

## 2 Methods

### 2.1 Study design, setting and population

Following an initial exploratory analysis, we conducted an interrupted time series regression of 14,224,908 attendances at consultant-led ED departments operated by 41 NHS Trusts in England between January 2019 and June 2020. Given our interest in the differential impacts of attendance rates by patient diagnosis, these 41 NHS Trusts were selected (from a population of 127 NHS Trusts that operate consultant-led EDs) because at least 75% of attendances at these Trusts had a valid electronic record of patients’ primary diagnosis in each month of the study period. 12 of the 41 NHS Trusts delivered major trauma services; there are 24 such organisations in England.

### 2.2 Variables and data sources

Anonymised extracts of the Accident and Emergency data set (AE) and its successor the Emergency Care Data Set (ECDS), were obtained from the National Commissioning Data Repository (NCDR) administered by NHS England [11,12]. These datasets are derived from real-time information systems maintained by clinical and administrative staff in NHS Trusts and are used to support service improvement, planning and research activities. Access to these datasets for individuals not employed by NHS England is controlled by NHS Digital. The extracts contained demographic, administrative and clinical information about all attendances at the selected ED departments in England between January 2019 and June 2020. The AE dataset was used for the period from January 2019 to March 2019 and ECDS from April 2019 to June 2020.

Attendances were counted by week (week 1 starting 1st January), year and by several demographic, socio-economic and clinical presentation variables: sex (male, female, other, not known), age group (0–19, 20–64, 65–74, 75+ years), ethnicity (white British, White Irish, White other, Indian, Pakistani, Bangladeshi, Asian other, Black Caribbean, Black African, Black other, Mixed – White and Black Caribbean, Mixed, White and Black African, Mixed – White and Asian, Mixed – other, Chinese, other ethnic group, not known, not stated), deprivation (deciles), urbanicity (urban, rural), arrival mode (by ambulance, self-conveyed), acuity (immediate/very urgent, urgent, standard/not urgent) and primary diagnosis (37 two-digit ED diagnoses classification code plus COVID-19).

Deprivation was defined using the English Indices of Deprivation 2015 [13]. This area-based measure was assigned based on the residence of the patient. Urbanicity was defined using the Rural and Urban Classification 2004 [14]. Patient diagnoses were mapped from SnomedCT codes recorded in ECDS [15].

### 2.3 Statistical methods

We used interrupted time series regression to estimate the size of the initial reduction in activity associated with the SARS-CoV-2 outbreak and lockdown policies and the rate of change in attendances following this interruption. The interruption was defined as the 4-week period from 11th March 2020 to 7th April 2020. Our primary analysis used linear regression with two main terms: week (a linear covariate), interruption (a binary covariate indicating whether the week was before or after the interruption) and an interaction term indicating the number of weeks since the interruption. A sensitivity analysis was conducted using negative binomial regression. A visual inspection of the trends alongside chronological information about the SARS-CoV-2 outbreak and lockdown measures were used to define the pre- and post-interruption periods.

The interrupted time series regression (linear model) was repeated for 75 subsets of the data defined by sex, age group, ethnicity, deprivation, urbanicity, arrival mode, acuity, and primary diagnosis.

Two metrics were derived from these models’ covariates.

%stepchangeatpointofinterruption=−p/(i+67t)

%activityincreasedperweekafterinterruption=(t+s)/(i+67t)

where

i is the model intercept
t is the model coefficient for the week number covariate
p is the model coefficient for the binary pre- post-interruption covariate
s is the model coefficient for the interaction term between binary pre- post-interruption and week number covariates

Note: week 1 is the first week of 2019 and week 67 is the first week after the interruption

Confidence intervals for these metrics were derived using Monte Carlo simulation with 10,000 replications using the model covariates and standard errors.

All analyses were undertaken using R v 4.0.2 [16].

Derived data supporting the findings of this study are available from the corresponding author on request.

### 2.4 Frameworks to guide interpretation of results

We use two frameworks to guide our approach. The first considers that patient decisions to attend ED which are influenced by three factors (i) symptom-related drivers such as anxiety and pain (ii) patient-related drivers such as coping capacity and feeling responsible for another's health and (iii) service-related drivers such as prior experience of ED, and availability of GP services [17]. The second framework classifies the factors that influence reductions in healthcare activity over the lockdown period: (i) the impact of policy choices (ii) changes in patient behaviour (iii) changes in the levels and types of morbidity [18]. The first framework is generic and longstanding, the second adds the COVID-19-pandemic context. We identify instances where our results appear to fit with or diverge from these frameworks.

## 3 Results

### 3.1 Characteristics of ED attendances and departments

Table 1 shows the demographic and socio-economic profile of the patient's attendances at EDs in the 41 selected NHS Trusts. Of the 14,224,908 attendances between January 2019 and June 2020, 50.8% were for women, 24.3% for people aged under 20 years, 77.5% for white people, 31.3% from the most deprived quintile of areas and 15.5% from rural areas. Attendances at the selected departments, with higher levels of diagnosis recording, had a broadly similar profile to those at all other departments, although white patients and patients from the most deprived areas were represented in marginally higher numbers.

**Table 1:** Characteristics of attendances at selected and other EDs, January 2019 to June 2020.

Grouping	Subgroup	EDs in 41 selected NHS Trusts			EDs in all 98 NHS Trusts where data was available*	
		Attendances		(%)	Attendances	(%)
total	total	14,224,908		(100.0%)	36,246,595	(100.0%)
sex	female	7,227,855		(50.8%)	18,468,247	(51.0%)
	male	6,994,849		(49.2%)	17,772,507	(49.0%)
	not known	2,204		(0.0%)	5,841	(0.0%)
age group	0	409,541		(2.9%)	1,153,670	(3.2%)
	1 to 4	987,480		(6.9%)	2,590,037	(7.1%)
	5 to 9	619,528		(4.4%)	1,584,453	(4.4%)
	10 to 14	664,382		(4.7%)	1,657,550	(4.6%)
	15 to 19	772,113		(5.4%)	1,934,219	(5.3%)
	20 to 64	7,083,774		(49.8%)	18,425,269	(50.8%)
	65 to 74	1,305,708		(9.2%)	3,181,321	(8.8%)
	75+	2,382,382		(16.7%)	5,720,076	(15.8%)
ethnic group	White	11,025,335		(77.5%)	26,527,430	(73.2%)
	Mixed	249,227		(1.8%)	677,992	(1.9%)
	Asian or Asian British	916,770		(6.4%)	2,588,251	(7.1%)
	Black or Black British	378,917		(2.7%)	1,468,464	(4.1%)
	Other ethnic groups	308,821		(2.2%)	1,301,763	(3.6%)
	not stated / not given	1,345,838		(9.5%)	3,682,695	(10.2%)
deprivation decile	1 - most deprived	2,527,138		(17.8%)	5,117,083	(14.1%)
	2	1,921,878		(13.5%)	4,665,253	(12.9%)
	3	1,608,064		(11.3%)	4,293,570	(11.8%)
	4	1,509,528		(10.6%)	3,761,878	(10.4%)
	5	1,347,702		(9.5%)	3,427,519	(9.5%)
	6	1,213,776		(8.5%)	3,198,845	(8.8%)
	7	1,165,595		(8.2%)	3,047,086	(8.4%)
	8	1,078,320		(7.6%)	2,962,980	(8.2%)
	9	964,930		(6.8%)	2,786,703	(7.7%)
	10 - least deprived	783,575		(5.5%)	2,600,837	(7.2%)
	not known	104,402		(0.7%)	384,841	(1.1%)
urbanicity	urban	11,937,002		(83.9%)	31,101,521	(85.8%)
	rural	2,208,835		(15.5%)	4,816,948	(13.3%)
	not known	79,071		(0.6%)	328,126	(0.9%)
acuity	immediate / very urgent	1,195,691		(8.4%)	2,871,363	(7.9%)
	urgent	4,356,997		(30.6%)	9,805,411	(27.1%)
	standard / not urgent	5,139,707		(36.1%)	11,537,165	(31.8%)
	not known	3,532,513		(24.8%)	12,032,656	(33.2%)
arrival mode	ambulance	4,652,537		(32.7%)	13,777,207	(38.0%)
	walk in	9,535,087		(67.0%)	29,280,390	(80.8%)
	not known	37,284		(0.3%)	2,185,125	(6.0%)
diagnosis	Laceration	749,439		(5.3%)	1,456,427	(4.0%)
	Contusion/abrasion	824,179		(5.8%)	1,705,865	(4.7%)
	Soft tissue inflammation	326,785		(2.3%)	627,137	(1.7%)
	Head injury	379,134		(2.7%)	778,237	(2.1%)
	Dislocation/fracture/joint injury/amputation	963,040		(6.8%)	1,982,033	(5.5%)
	Sprain/ligament injury	841,864		(5.9%)	1,682,751	(4.6%)
	Muscle/tendon injury	255,103		(1.8%)	536,649	(1.5%)
	Burns and scalds	93,226		(0.7%)	189,633	(0.5%)
	Foreign body	120,954		(0.9%)	255,916	(0.7%)
	Poisoning (incl overdose)	371,353		(2.6%)	727,118	(2.0%)
	Visceral injury	20,852		(0.1%)	69,280	(0.2%)
	Infectious disease	258,491		(1.8%)	579,305	(1.6%)
	Local infection	228,471		(1.6%)	486,567	(1.3%)
	Septicaemia	126,727		(0.9%)	254,980	(0.7%)
	Cardiac conditions	776,126		(5.5%)	1,442,157	(4.0%)
	Cerebro-vasc. conditions	288,890		(2.0%)	546,554	(1.5%)
	Other vascular conditions	81,501		(0.6%)	165,185	(0.5%)
	Haematological conditns	123,146		(0.9%)	256,013	(0.7%)
	CNS conditions	248,961		(1.8%)	468,512	(1.3%)
	Respiratory conditions	1,220,562		(8.6%)	2,465,239	(6.8%)
	SARS-CoV-2	32,269		(0.2%)	58,857	(0.2%)
	Gastrointestinal conditns	1,069,989		(7.5%)	2,126,852	(5.9%)
	Urological conditions	615,140		(4.3%)	1,267,216	(3.5%)
	Obstetric conditions	10,530		(0.1%)	19,889	(0.1%)
	Gynaecological conditns	208,364		(1.5%)	424,854	(1.2%)
	Endocrinological conditns	104,292		(0.7%)	207,478	(0.6%)
	Dermatological conditns	106,779		(0.8%)	252,694	(0.7%)
	Allergy	73,322		(0.5%)	183,220	(0.5%)
	Facio-maxillary conditns	70,177		(0.5%)	138,417	(0.4%)
	ENT conditns	319,143		(2.2%)	710,668	(2.0%)
	Psychiatric conditns	311,973		(2.2%)	601,324	(1.7%)
	Ophthalmological condns	252,768		(1.8%)	622,251	(1.7%)
	Social problem	63,504		(0.4%)	125,283	(0.3%)
	Other injury	4,087		(0.0%)	9,642	(0.0%)
	Diagnosis not classifiable	908,051		(6.4%)	2,661,728	(7.3%)
	Nothing abnormal detected	1,182,251		(8.3%)	2,165,171	(6.0%)
	not known	593,465		(4.2%)	7,995,493	(22.1%)

⁎ This is itself a subset of all 127 NHS Trusts that operate consultant-led EDs.

### 3.2 **COVID-19 related attendances**

Attendances of patients with suspected or confirmed COVID-19, peaked in the first week of April. During this week, attendances for COVID-19represented 5.6% of all attendances at the study sites. These attendances reduced rapidly and by the last week of June, made up less than half of one percent of attendances. The recording of confirmed or suspected SARS-CoV-2 infections may not have been complete, particularly at the start of the outbreak.

### 3.3 Interrupted time series regression

**Fig. 1:**
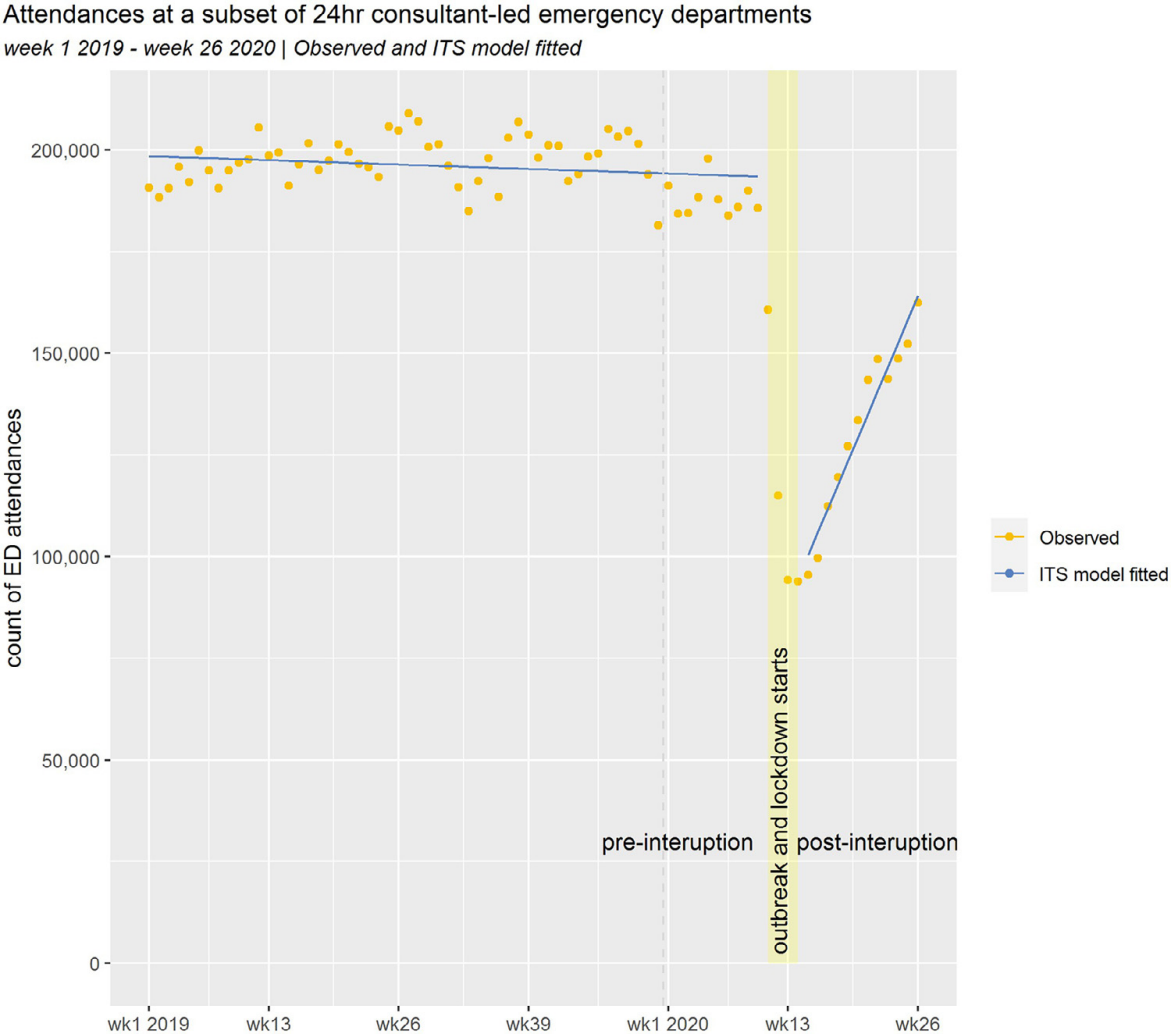
Attendances at a subset of 24 h consultant-led emergency departments Week 1 2019 – week 26 2020 | Observed and ITS model fitted.

Fig. 1 shows the number of ED attendances at the selected emergency departments by week from the beginning 2019 to week 26 in 2020. Prior to the SARS_Cov_2 outbreak, attendance rates varied from 181,500 to 209,100 per week. Some seasonal variation appears to be present. Between weeks 11 and 14, (2020), the number of attendances fell rapidly to a low of 93,800 attendances per week. From week 15 in 2020, attendance rates started to increase and by week 26, there were 162,400 attendances.

The fitted values from our linear interrupted time series model are also shown on Fig. 1. The prior and post interruption trends are broadly linear, and all model coefficients were statistically significant (see Table 2). The model indicated that the interruption was associated with a 51.1% reduction (95% CI 46.3–55.9%) in ED attendances. Following this initial shock, activity increased linearly by 3.0% per week (95% CI 2.5–3.5%), as measured from the pre-outbreak level.  We fitted a negative binomial model as a sensitivity analysis. The results from the two models were consistent (see supplementary tables 4 and 5).

**Table 2:** Covariates and coefficients of the linear interrupted time series regression.

Term	Estimate	Standard error	*p* value
(intercept)	198,577.6	1646.5	<0.0005
week number	−82.3	45.4	0.074
interruption (binary)	−98,616.1	4319.2	<0.0005
week number * interruption	5883.0	537.5	<0.0005

### 3.4 Subgroup analyses

**Fig. 2:**
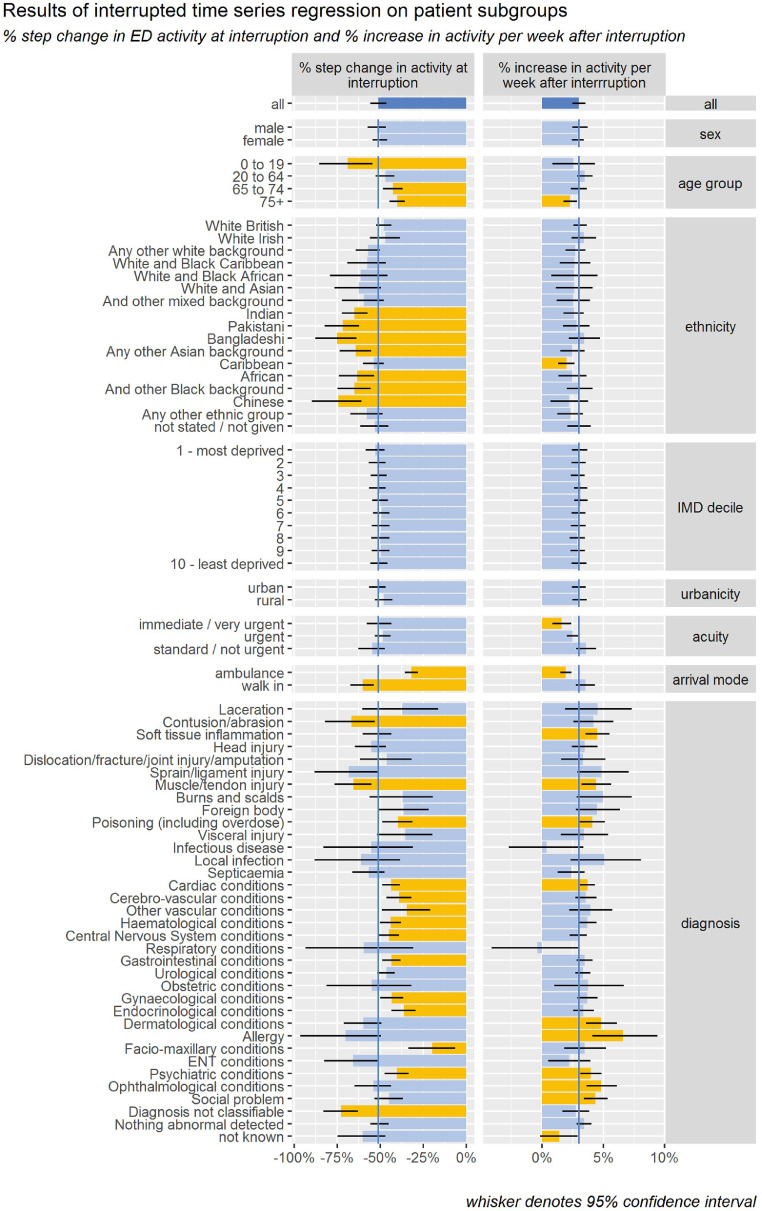
Results of interrupted time series regression on patient subgroups % step change in ED activity at interruption and % increase in activity per week after interruption.

Fig. 2 and Table 3 show the results of fitting linear interrupted time series regression models to 75 subgroups defined by sex, age, ethnicity, deprivation, urbanicity, arrival mode, acuity, and diagnosis. The initial reduction and post-interruption trend for subgroups defined by age, deprivation, urbanicity and acuity, did not vary significantly from those of attendances as a whole. However significant differences were noted for some of the subgroups defined by age, ethnicity, arrival mode and diagnosis. Significantly larger initial reductions were seen in those aged 0–19 years (69.1%) those of South Asian (68.8%), Black (61.4%), and Chinese (74.5%) ethnicities, those that self-conveyed (60.3%) and those presenting with contusions or abrasions (66.9%), muscle and tendon injuries (65.6%), and those with a diagnosis that was not classifiable (72.7%). Significantly smaller initial reductions were seen in those aged 65–74 years (42.6%), 75+ years (40.1%), those conveyed by ambulance (31.9%), and those presenting with poisoning (39.7%), cardiac (43.7%), cerebro-vascular (39.0%), other vascular (34.6%), haematological (44.0%), CNS (44.9%), gastrointestinal (43.4%), gynaecological (43.2%), endocrinological (36.1%), maxillo-facial (19.7%) and psychiatric (40.4%) conditions. The post-outbreak rate of increase in attendances per week was significantly faster for those presenting with soft tissue inflammation (4.5%), muscle and tendon injury (4.4%), dermatological conditions (4.8%), allergies (6.6%) and psychiatric conditions (4.0%), ophthalmological conditions (4.8%) and social problems (4.4%) and significantly slower for those aged 75+ years (2.3%), of Caribbean ethnicity (2.0%), for those conveyed by ambulance (1.9%) and for the most urgent cases (1.6%).

**Table 3:** Results of interrupted time series regression on patient subgroups.

		% step change in activity at interruption				% increase in activity per week after interruption			
Characteristic	Subgroup	%		95% CI		%		95% CI	
All	All	51.1%		46.4%	55.7%	3.0%		2.5%	3.6%
Sex	male	51.9%		46.5%	57.4%	3.1%		2.4%	3.7%
	female	50.2%		45.9%	54.5%	2.9%		2.4%	3.4%
age group	0 to 19	69.1%		54.6%	85.7%	2.6%		0.9%	4.3%
	20 to 64	47.1%		41.7%	52.8%	3.5%		2.8%	4.1%
	65 to 74	42.6%		37.1%	48.3%	3.0%		2.3%	3.7%
	75+	40.1%		35.6%	44.7%	2.3%		1.8%	2.8%
ethnicity	White British	47.9%		43.3%	52.6%	3.1%		2.6%	3.6%
	White Irish	47.0%		38.7%	55.8%	3.4%		2.4%	4.4%
	Any other white background	57.1%		50.1%	64.4%	2.7%		1.9%	3.5%
	White and Black Caribbean	57.6%		47.1%	68.8%	2.7%		1.4%	3.9%
	White and Black African	61.3%		45.6%	78.9%	2.6%		0.8%	4.5%
	White and Asian	62.6%		49.9%	76.9%	2.6%		1.1%	4.1%
	And other mixed background	59.6%		47.6%	72.0%	2.5%		1.2%	3.9%
	Indian	64.9%		57.8%	72.5%	2.6%		1.8%	3.4%
	Pakistani	71.8%		62.3%	82.0%	2.8%		1.7%	3.9%
	Bangladeshi	75.3%		64.0%	87.9%	3.4%		2.2%	4.7%
	Any other Asian background	64.3%		55.4%	73.5%	2.5%		1.4%	3.5%
	Caribbean	53.9%		48.0%	59.9%	2.0%		1.3%	2.7%
	African	63.5%		53.8%	73.9%	2.5%		1.4%	3.6%
	And other Black background	65.1%		55.9%	74.9%	3.1%		2.0%	4.2%
	Chinese	74.5%		60.8%	89.3%	2.2%		0.7%	3.8%
	Any other ethnic group	57.8%		49.0%	67.3%	2.3%		1.3%	3.4%
	not stated / not given	53.2%		45.2%	61.8%	3.0%		2.1%	4.0%
deprivation	1 - most deprived	52.9%		47.6%	58.4%	3.1%		2.4%	3.7%
	2	51.8%		46.9%	56.7%	3.0%		2.4%	3.6%
	3	50.9%		46.1%	55.7%	2.9%		2.4%	3.5%
	4	51.6%		46.9%	56.3%	3.2%		2.6%	3.7%
	5	50.0%		45.4%	54.8%	3.2%		2.6%	3.7%
	6	49.4%		44.6%	54.2%	3.0%		2.4%	3.5%
	7	49.8%		44.7%	55.0%	2.9%		2.4%	3.5%
	8	49.9%		44.6%	55.4%	2.9%		2.3%	3.5%
	9	49.9%		44.9%	55.0%	2.9%		2.3%	3.5%
	10 - least deprived	50.9%		45.8%	56.0%	3.0%		2.4%	3.6%
urbanicity	urban	51.6%		46.9%	56.5%	3.0%		2.5%	3.6%
	rural	47.9%		42.9%	53.0%	3.1%		2.5%	3.7%
acuity	immediate / very urgent	50.4%		43.6%	57.8%	1.6%		0.8%	2.4%
	urgent	48.4%		44.0%	53.1%	2.5%		2.0%	3.0%
	standard / not urgent	54.8%		47.4%	62.9%	3.6%		2.8%	4.4%
arrival mode	ambulance	31.9%		28.2%	35.7%	1.9%		1.5%	2.4%
	walk in	60.3%		53.5%	67.6%	3.5%		2.7%	4.3%
diagnosis	Laceration	37.3%		16.5%	60.9%	4.5%		2.0%	7.3%
	Contusion/abrasion	66.9%		53.1%	82.1%	4.2%		2.6%	5.9%
	Soft tissue inflammation	51.5%		43.2%	60.2%	4.5%		3.5%	5.5%
	Head injury	55.5%		46.6%	64.8%	3.5%		2.4%	4.5%
	Dislocation/fracture/joint injury/amputation	46.3%		31.9%	61.6%	3.3%		1.6%	5.1%
	Sprain/ligament injury	68.4%		50.4%	87.9%	4.9%		2.8%	7.0%
	Muscle/tendon injury	65.6%		55.0%	76.7%	4.4%		3.2%	5.7%
	Burns and scalds	37.0%		19.3%	55.7%	5.0%		2.8%	7.3%
	Foreign body	36.3%		21.8%	51.4%	4.5%		2.7%	6.4%
	Poisoning (including overdose)	39.7%		31.1%	48.6%	4.1%		3.0%	5.2%
	Visceral injury	35.6%		19.9%	52.3%	3.4%		1.5%	5.4%
	Infectious disease	55.4%		31.5%	84.7%	0.4%		-2.6%	3.3%
	Local infection	61.1%		38.6%	87.8%	5.1%		2.3%	8.1%
	Septicaemia	56.7%		47.6%	66.5%	2.4%		1.3%	3.5%
	Cardiac conditions	43.7%		38.7%	48.9%	3.7%		3.1%	4.3%
	Cerebro-vascular conditions	39.0%		31.9%	46.4%	3.6%		2.7%	4.4%
	Other vascular conditions	34.6%		20.6%	49.3%	3.9%		2.2%	5.7%
	Haematological conditions	44.0%		37.7%	50.1%	3.7%		3.0%	4.4%
	CNS conditions	44.9%		39.1%	51.1%	3.0%		2.2%	3.7%
	Respiratory conditions	59.7%		30.0%	94.9%	-0.4%		-4.1%	3.1%
	Gastrointestinal conditions	43.4%		38.0%	48.9%	3.5%		2.8%	4.1%
	Urological conditions	46.4%		41.5%	51.6%	3.3%		2.7%	3.9%
	Obstetric conditions	54.9%		32.3%	81.5%	3.7%		1.0%	6.7%
	Gynaecological conditions	43.3%		36.6%	50.1%	3.7%		2.9%	4.5%
	Endocrinological conditions	36.2%		29.3%	43.4%	3.4%		2.5%	4.2%
	Dermatological conditions	59.9%		49.2%	71.2%	4.8%		3.6%	6.1%
	Allergy	70.3%		48.9%	95.4%	6.6%		4.1%	9.4%
	Facio-maxillary conditions	19.7%		6.4%	33.4%	3.5%		1.8%	5.2%
	ENT conditions	65.9%		51.0%	82.5%	2.2%		0.5%	4.0%
	Psychiatric conditions	40.4%		33.5%	47.7%	4.0%		3.1%	4.8%
	Ophthalmological conditions	54.0%		43.5%	64.9%	4.8%		3.6%	6.1%
	Social problem	44.9%		36.8%	53.4%	4.4%		3.4%	5.3%
	Diagnosis not classifiable	72.7%		62.9%	83.4%	2.7%		1.6%	3.9%
	Nothing abnormal detected	50.3%		45.0%	55.7%	3.4%		2.8%	4.0%
	not known	60.2%		47.3%	74.6%	1.4%		−0.2%	3.0%

% step change in ED activity at interruption and % increase in activity per week after interruption.

## 4 Discussion

### 4.1 Summary of main findings

We examined the impact of SARS-CoV-2 and the associated lockdown measures on levels of ED activity in England. We found that attendance rates halved almost immediately after lockdown was announced. The absolute variation across a broad suite of attendance subsets was relatively small. For example, the interquartile range for the initial step change in activity across 35 diagnoses was 40.0–60.0%. Thus, the initial reductions were large and relatively uniform. The reduction varied by age groups, ethnicity, arrival mode and diagnostic group but not by sex, deprivation, urbanicity or acuity. We also found that attendance rates began to increase 2–3 weeks after the lockdown was announced and have increased in a linear fashion up to the end of June 2020.

Activity reductions immediately after lockdown were larger in children and young people than in older adults. This might be explained in part by reductions in childhood illnesses and accidents as the lockdown limited opportunities for outdoor play, sporting activities and for the transmission of childhood infections. Prior to the pandemic, children and young people were more likely than adults to use ED inappropriately [19]. O'Cathain et al suggest that clinically unnecessary ED visits in children are sometimes caused by parents’ fear of the consequences of failing to bring their child to ED [17]. The countervailing risk of SARS-CoV-2 infection may have acted to offset this tendency, reducing the level of clinically unnecessary ED visits in children. We note however that all-age attendances where no clinical problem was detected, reduced and subsequently increased at a very similar rate to ED attendances as a whole.

Our frameworks might lead us to expect larger reduction in the less acute or urgent presentations as thresholds for referrals from GPs, 999 and 111 increased, but we found no significant differences in the rates of activity reduction or subsequent increase by acuity level. More granular analysis is required to explain these results. It may be for example that large reductions in the most urgent injury presentations (e.g., RTA) are offset by smaller reductions in the most urgent illness presentations (e.g., stroke, AMI) or vice versa. The acuity field was introduced with the ECDS dataset in recent years and as such assessments of the quality and consistency of acuity coding may also be warranted.

Ambulance-conveyed ED attendances fell at approximately half the rate as walk-in attendances. Ambulance-conveyed attendances tend to be more complex and acute than walk-in attendances. It might be tempting therefore to attribute greater reductions in walk-in attendances to the fact that these attendances are more amenable to delay or self-care. However, as noted above, we observed no significant differences in ED activity reductions between acuity levels. Alternatively, this effect may be attributable to changes in patient behaviour, with patients more likely to call an ambulance during the lockdown period as public transport and taxi services were limited and as a means of accessing healthcare advice without leaving home. We note that the proportion of 999-calls that did not result in a conveyance to ED increased substantially during lockdown [18]; indicating a change in system-behaviour. The effect may also be a function of the differential activity reduction by age. Older people are more likely to be conveyed by ambulance and reductions in attendances for older people were smaller in relative terms than those seen in children. This is another instance where more granular analysis, by age and conveyance method, may be helpful.

Attendances for contusions, abrasions, muscle and tendon injuries- reduced at significantly greater rate than other forms of attendance. This might be explained by a reduction in workplace injuries (as many workers were furloughed), injuries that would have been sustained during sporting and outdoor play activities and injuries associated with alcohol consumption in public places. Attendances for a range of more complex medical conditions (cardiac, cerebro-vascular, other vascular, haematological, CNS, gastrointestinal) also fell substantially, but to a lesser extent than many types of injury presentation. In many cases the underlying conditions that precipitated these attendances would have pre-dated the lockdown period, such that there was only limited opportunity for behaviour changes during lockdown to reduce the risk of an adverse health event. Attendances for psychiatric conditions and for poisonings (which include overdoses) fell at a slower rate immediately after lockdown and these attendances along with those for social problems have subsequently increased more rapidly. Several studies have reported increases in psychiatric morbidity during the lockdown period [20], [21], [22].

We found no significant differences in initial activity reductions or subsequent activity increases rates by sex. The combination of the different potential drivers to change levels of ED attendances (i.e., policy/system, behaviour, incidence/morbidity) during this time seem to have affected both males and females equally. This may or may not hold for each individual component.

We similarly note no difference in initial activity reductions or subsequent activity increases rates by rurality / urbanicity. Our frameworks here might have indicated greater reductions in urban areas than in rural areas, since travel to ED, an indirect cost to patients attending ED, might be lower in these areas, increasing the likelihood of clinically unnecessary attendances. But this does not appear to be the case. If urban and rural public transport systems were affected differentially then this does not appear to have had a substantial differential impact on ED attendances rates.

Given that we observed no differences in initial activity reductions or subsequent activity increases rates by rurality / urbanicity, it is not surprising that we also found no difference by deprivation decile of residence. The lockdown rules affected people across all socio-economic groups and the take-up of the Government job retention (furlough) scheme does not appear to follow the usual patterns of deprivation and affluence [23].

Initial reductions in ED attendances were significantly greater amongst people from Asian or Asian British and Black or Black British backgrounds than for white people, and subsequent activity increases were slower (although not significantly so). Early anecdotal reports of increased risks of hospitalisation and mortality amongst Asian and Black people have been confirmed by subsequent research [24,25]. Other reports have suggested that aspects of the algorithms used by NHS111 to assess the severity of COVID-19 symptoms may not be appropriate for Black people [26]. A recent study estimated that 60% of Black people in the UK do not feel their health is as well protected by the NHS compared to white people [27].

### 4.2 Relationship with existing literature

Mafham et al. [28] examined the impact of the covid-19 pandemic on admission rates for and management of acute coronary syndromes (ACS) in England. They found a 40% reduction in admissions per week with parallel reductions of 21% to 37% in associated revascularisation procedures along with a reduction in hospital stay (median 4 days to 3 days). The decline in admissions, which began before lockdown (23 March 2020), was partly reversed during April and May 2020, such that by the last week of May 2020, the numbers of admission represented a 16% reduction from baseline. They concluded that the reduced number of admissions “…is likely to have resulted in increases in out-of-hospital deaths and long-term complications of myocardial infarction and missed opportunities to offer secondary prevention treatment for patients with coronary heart disease.” Transcatheter and Surgical Treatment of Severe Aortic Stenosis[29] activity decreased significantly in England following the SARS-CoV-2 outbreak. Similar reductions in hospital admissions for ACS in Italy [30] and myocardial infarction were observed in France [31].

Isba et al (2020) reported that paediatric ED attendances reduced by 30.4% and 33.8% at two hospitals in Greater Manchester in March 2020 compared with March 2019 [32]. Periera Gray et al (2020) found that face to face GP consultations fell by 92.5% in the second half of March 2020 whereas the number of telephone consultations increased by 85.6% [33]. McDonald et al (2020) report that childhood immunisations reduced by 19.8% in the three weeks after social-distancing measures were introduced in England [34].

### 4.3 Limitations of the study

The time period defined as the interruption in our analysis was assigned informally through a visual inspection of the time series and with reference to the timing of key events such as the UK Government's lockdown announcement. Alternative time definitions for the interruption may result in subtly different effect estimates. Methods such as change-point detection could be used to define the interruption and whilst these may increase analytical reproducibility and transparency, they are nonetheless subject their own limitations.

Our analysis is based on a selection of ED sites in England whose recording levels meet specified criteria. This selection process is a potential source of bias. However, a comparison of attendance characteristics between the study sites and all other sites, suggests that the study sites are broadly representative.

Our analysis fits a linear regression model to the observed time series. A more sophisticated modelling method, negative binomial regression, provided very similar results. We might expect some seasonal variation in attendance rates, but our analysis made no attempt to adjust for this variation. This manifests as wider confidence intervals around our effect sizes. We would expect a seasonally adjusted model to detect differences in an increased number of activity subgroups.

Our study is a series of unadjusted analyses, exploring differences by gender, age groups, ethnicity etc in turn. Effects observed in one variable may therefore be an expression of effects driven by another correlated variable. A multivariable approach, which adjusts for several variables simultaneously may allow the effects of individual variables to be teased out explicitly. The formulation of such as approach is not without its challenges.

Whilst our analysis explores the differential impacts of the SARS-Cov-2 pandemic and associated lockdown measures across a large number of population and diagnostic subgroups, it is likely there are heterogenous effects within these subgroups.

### 4.4 Policy or practice implications

The NHS advises patients attend major ED departments for ‘genuine life-threatening conditions’[35] suggesting that less severe conditions can be treated elsewhere. Attendances at major EDs grew at an average of 1.3% per annum between 2010 and 2019 [36]. Strategies to mediate the growth in demand have often focused on reducing the number of clinically unnecessary attendances. At the start of the lockdown the NHS advised EDs to remain open, with special measures to deal with COVID-19 patients and infection risks [37]. In the short term the frequency of life-threatening conditions might be regarded as somewhat immutable, yet our analysis suggests attendance rates halved almost immediately after lockdown was announced. We note that these reductions were seen alongside falls in many other services such as GP consultations and walk-in centres [38]. In March 2020, calls to NHS 111 were running at twice the usual rate, but 38.7% of these calls were abandoned after waiting more than 30 s [39].

The substantial reduction in ED activity after lockdown and the comparatively slow rates at which activity has subsequently increased, pose two important questions. Do the activity reductions: (i) represent missed opportunities to treat conditions leading to avoidable morbidity and mortality and/or (ii) imply that many ED attendances that occur in usual circumstances are clinically unnecessary. The task of those responsible for policy development and service design will be to identify the circumstances where these two situations apply so as to minimise avoidable morbidity and mortality and unnecessary ED attendances.

Our research provides little direct evidence of the notion that a substantial proportion of ED attendances are clinically unnecessary. We found no differences in the rates of reduction in activity following the lockdown between acuity levels, rurality / urbanicity or in cases where nothing abnormal was detected. The proposed ‘call before you walk’ initiative may help with ED crowding by reducing some of the peaks and troughs in demand but its potential impact on reducing the total volumes of attendances is far from clear and if implemented, it should be subject to robust evaluation. The initiative provides a fresh opportunity to review GP, 999, and 111 referral thresholds and ambulance conveyance thresholds.

Given these findings, the scale of the reductions in ED attendances of children and Asian and Black patients is a cause for concern. Although smaller in scale, the reduction in complex illness presentations is a concern also. The decision whether to attend ED during the COVID pandemic represents a balance of risks; the risk of failing to treat an important health condition versus the risks of COVID infection or transmission. Healthcare systems should consider how they support these patient groups to calibrate their decisions against the true risks. Targeted communications may be required.

The UK Government have adjusted the nature and extent of non-pharmaceutical interventions several times since the initial lockdown was introduced. These interventions appear set to remain in place in some form through the forthcoming winter period. The most recently published figures suggest that by October 2020 ED attendance rates had still not returned to pre-lockdown levels [40].

## 5 Conclusions

The COVID-19 pandemic and associated lockdown measures led to substantially fewer attendances to ED. Activity remains below pre-pandemic levels. These findings raise concern about excess avoidable morbidity and mortality during the pandemic especially in the event of future waves of infection and associated non-pharmaceutical countermeasures. Further research to identify and monitor these adverse effects is warranted.

## Authors contributions

SW conceived of and conducted the analysis. MM advised on methods, reviewed the analysis. SW and MM drafted the manuscript All authors contributed to the discussion section, reviewed and commented on the draft manuscript.

## Ethics approval

Ethnic approval not required. This study was retrospective analysis of the impact of a disease outbreak and involved no intervention. The data used were routinely collected and non-identifiable.

## Declaration of Competing Interest

No conflicts of interest to declare

## Appendix Supplementary materials

Image, application 1

Image, application 2
