# Supplementary material for: Impact of the SARS-CoV-2 pandemic and associated lockdown measures on attendances at emergency departments in English hospitals: A retrospective database study
Source: Lancet Reg Health Eur. 2021 Jan 13;2:100034. doi: 10.1016/j.lanepe.2021.100034 (PMC7837109; doi:10.1016/j.lanepe.2021.100034)
Supplement: Supplementary file 1 [file mmc1.docx]

Supplementary table 4: Covariates and coefficients of the negative binomial interrupted time series regression

| **term** | **estimate** | **IRR** | **standard error** | **p value** |
| --- | --- | --- | --- | --- |
| (intercept) | 12.1992 | 198,632.3 | 0.0091 | <0.0005 |
| week number | -0.0004 | 0.9996 | 0.0003 | .0091 |
| interruption (binary) | -0.6875 | 0.5028 | 0.0240 | <0.0005 |
| week number * interruption | 0.0461 | 1.0472 | 0.0030 | <0.0005 |
