# Supplementary material for: Impact of the SARS-CoV-2 pandemic and associated lockdown measures on attendances at emergency departments in English hospitals: A retrospective database study
Source: Lancet Reg Health Eur. 2021 Jan 13;2:100034. doi: 10.1016/j.lanepe.2021.100034 (PMC7837109; doi:10.1016/j.lanepe.2021.100034)
Supplement: Supplementary file 2 [file mmc2.docx]

Supplementary table 5: Observed and ITS fitted values, linear and negative binomial models

|  | **ED attendances per week** | | | **Relative differences** | |
| --- | --- | --- | --- | --- | --- |
| **year - week** | **observed** | **fitted**  **(linear model)** | **fitted**  **(negative binomial model)** | **observed to fitted**  **(linear model)** | **observed to fitted (negative binomial model)** |
| 2019 - week 1 | 190,673 | 198,495 | 198,548 | 4.1% | 4.1% |
| 2019 - week 2 | 188,295 | 198,413 | 198,463 | 5.4% | 5.4% |
| 2019 - week 3 | 190,652 | 198,331 | 198,378 | 4.0% | 4.1% |
| 2019 - week 4 | 195,866 | 198,248 | 198,293 | 1.2% | 1.2% |
| 2019 - week 5 | 192,137 | 198,166 | 198,209 | 3.1% | 3.2% |
| 2019 - week 6 | 199,868 | 198,084 | 198,124 | -0.9% | -0.9% |
| 2019 - week 7 | 194,958 | 198,001 | 198,040 | 1.6% | 1.6% |
| 2019 - week 8 | 190,568 | 197,919 | 197,955 | 3.9% | 3.9% |
| 2019 - week 9 | 194,971 | 197,837 | 197,870 | 1.5% | 1.5% |
| 2019 - week 10 | 196,887 | 197,754 | 197,786 | 0.4% | 0.5% |
| 2019 - week 11 | 197,737 | 197,672 | 197,702 | 0.0% | 0.0% |
| 2019 - week 12 | 205,522 | 197,590 | 197,617 | -3.9% | -3.8% |
| 2019 - week 13 | 198,572 | 197,507 | 197,533 | -0.5% | -0.5% |
| 2019 - week 14 | 199,412 | 197,425 | 197,449 | -1.0% | -1.0% |
| 2019 - week 15 | 191,191 | 197,343 | 197,364 | 3.2% | 3.2% |
| 2019 - week 16 | 196,485 | 197,260 | 197,280 | 0.4% | 0.4% |
| 2019 - week 17 | 201,611 | 197,178 | 197,196 | -2.2% | -2.2% |
| 2019 - week 18 | 195,055 | 197,096 | 197,112 | 1.0% | 1.1% |
| 2019 - week 19 | 197,400 | 197,013 | 197,027 | -0.2% | -0.2% |
| 2019 - week 20 | 201,406 | 196,931 | 196,943 | -2.2% | -2.2% |
| 2019 - week 21 | 199,474 | 196,849 | 196,859 | -1.3% | -1.3% |
| 2019 - week 22 | 196,560 | 196,766 | 196,775 | 0.1% | 0.1% |
| 2019 - week 23 | 195,670 | 196,684 | 196,691 | 0.5% | 0.5% |
| 2019 - week 24 | 193,360 | 196,602 | 196,607 | 1.7% | 1.7% |
| 2019 - week 25 | 205,690 | 196,519 | 196,523 | -4.5% | -4.5% |
| 2019 - week 26 | 204,777 | 196,437 | 196,439 | -4.1% | -4.1% |
| 2019 - week 27 | 209,052 | 196,355 | 196,356 | -6.1% | -6.1% |
| 2019 - week 28 | 207,035 | 196,272 | 196,272 | -5.2% | -5.2% |
| 2019 - week 29 | 200,745 | 196,190 | 196,188 | -2.3% | -2.3% |
| 2019 - week 30 | 201,395 | 196,108 | 196,104 | -2.6% | -2.6% |
| 2019 - week 31 | 196,108 | 196,026 | 196,021 | 0.0% | 0.0% |
| 2019 - week 32 | 190,902 | 195,943 | 195,937 | 2.6% | 2.6% |
| 2019 - week 33 | 184,981 | 195,861 | 195,853 | 5.9% | 5.9% |
| 2019 - week 34 | 192,362 | 195,779 | 195,770 | 1.8% | 1.8% |
| 2019 - week 35 | 197,973 | 195,696 | 195,686 | -1.2% | -1.2% |
| 2019 - week 36 | 188,486 | 195,614 | 195,603 | 3.8% | 3.8% |
| 2019 - week 37 | 202,973 | 195,532 | 195,519 | -3.7% | -3.7% |
| 2019 - week 38 | 206,883 | 195,449 | 195,436 | -5.5% | -5.5% |
| 2019 - week 39 | 203,772 | 195,367 | 195,352 | -4.1% | -4.1% |
| 2019 - week 40 | 198,153 | 195,285 | 195,269 | -1.4% | -1.5% |
| 2019 - week 41 | 201,112 | 195,202 | 195,185 | -2.9% | -2.9% |
| 2019 - week 42 | 201,021 | 195,120 | 195,102 | -2.9% | -2.9% |
| 2019 - week 43 | 192,346 | 195,038 | 195,019 | 1.4% | 1.4% |
| 2019 - week 44 | 194,128 | 194,955 | 194,936 | 0.4% | 0.4% |
| 2019 - week 45 | 198,339 | 194,873 | 194,852 | -1.7% | -1.8% |
| 2019 - week 46 | 199,094 | 194,791 | 194,769 | -2.2% | -2.2% |
| 2019 - week 47 | 205,152 | 194,708 | 194,686 | -5.1% | -5.1% |
| 2019 - week 48 | 203,190 | 194,626 | 194,603 | -4.2% | -4.2% |
| 2019 - week 49 | 204,662 | 194,544 | 194,520 | -4.9% | -5.0% |
| 2019 - week 50 | 201,489 | 194,461 | 194,437 | -3.5% | -3.5% |
| 2019 - week 51 | 193,920 | 194,379 | 194,354 | 0.2% | 0.2% |
| 2019 - week 52 | 181,512 | 194,297 | 194,271 | 7.0% | 7.0% |
| 2020 - week 1 | 191,260 | 194,214 | 194,188 | 1.5% | 1.5% |
| 2020 - week 2 | 184,283 | 194,132 | 194,105 | 5.3% | 5.3% |
| 2020 - week 3 | 184,442 | 194,050 | 194,022 | 5.2% | 5.2% |
| 2020 - week 4 | 188,329 | 193,967 | 193,939 | 3.0% | 3.0% |
| 2020 - week 5 | 197,860 | 193,885 | 193,857 | -2.0% | -2.0% |
| 2020 - week 6 | 187,895 | 193,803 | 193,774 | 3.1% | 3.1% |
| 2020 - week 7 | 183,816 | 193,720 | 193,691 | 5.4% | 5.4% |
| 2020 - week 8 | 185,932 | 193,638 | 193,608 | 4.1% | 4.1% |
| 2020 - week 9 | 189,910 | 193,556 | 193,526 | 1.9% | 1.9% |
| 2020 - week 10 | 185,720 | 193,473 | 193,443 | 4.2% | 4.2% |
| 2020 - week 11 | 160,695 | interruption | | | |
| 2020 - week 12 | 115,045 |  |  |  |  |
| 2020 - week 13 | 94,283 |  |  |  |  |
| 2020 - week 14 | 93,846 |  |  |  |  |
| 2020 - week 15 | 95,445 | 100,329 | 101,641 | 5.1% | 6.5% |
| 2020 - week 16 | 99,688 | 106,129 | 106,390 | 6.5% | 6.7% |
| 2020 - week 17 | 112,444 | 111,930 | 111,361 | -0.5% | -1.0% |
| 2020 - week 18 | 119,527 | 117,731 | 116,564 | -1.5% | -2.5% |
| 2020 - week 19 | 127,127 | 123,531 | 122,010 | -2.8% | -4.0% |
| 2020 - week 20 | 133,495 | 129,332 | 127,711 | -3.1% | -4.3% |
| 2020 - week 21 | 143,363 | 135,133 | 133,678 | -5.7% | -6.8% |
| 2020 - week 22 | 148,572 | 140,933 | 139,923 | -5.1% | -5.8% |
| 2020 - week 23 | 143,725 | 146,734 | 146,461 | 2.1% | 1.9% |
| 2020 - week 24 | 148,732 | 152,535 | 153,304 | 2.6% | 3.1% |
| 2020 - week 25 | 152,246 | 158,335 | 160,467 | 4.0% | 5.4% |
| 2020 - week 26 | 162423 | 164,136 | 167,964 | 1.1% | 3.4% |
